# Hepatitis B Infection among high risk population: a seroepidemiological survey in Southwest of Iran

**DOI:** 10.1186/1471-2334-12-378

**Published:** 2012-12-27

**Authors:** Abdolmajid Khosravani, Bahador Sarkari, Halimeh Negahban, Asghar Sharifi, Mehdi Akbartabar Toori, Owrang Eilami

**Affiliations:** 1 Faculty of Medicine, Yasouj University of Medical Sciences, Yasouj, Iran; 2 Basic Sciences in Infectious Diseases Research center, Shiraz University of Medical Sciences, Shiraz, Iran; 3 Social Determinants of Health Research Center, Yasouj University of Medical Sciences, Yasouj, Iran

**Keywords:** Seroprevalence, HBV, High risk group, Prevalence, Iran

## Abstract

**Background:**

Hepatitis B virus (HBV) infection remains a major global health problem. This study aimed to assess the prevalence and risk behaviors for HBV infection among high risk groups in Kohgiloyeh and Boyerahmad province, in Southwest of Iran.

**Methods:**

Blood samples were collected from 2009 subjects, between 2009 and 2010 in Kohgiloyeh and Boyerahmad province, in southwest of Iran. Recruited subjects were the high risk groups for HBV infection, including inmates, injecting drug users, health care workers, patients on maintenance haemodialysis, hemophilic patients and patients with a history of blood transfusion. Their serum samples were tested for the presence of antibodies to hepatitis B core antigen (HBc IgM, IgG) by enzyme-linked immunosorbent assay (ELISA). Seropositive specimens were tested for HBsAg. Demographic features of participants were recorded during sample collecting.

**Results:**

HBsAg was detected in 24 of the 2009 subjects, giving an overall prevalence of 1.2%. All HBsAg positive cases were males. The prevalence of HBsAg among injection drug users was 3.2%. Significant correlation was found between HBV infection and drug abuse, level of education and place of residence (p<0.05), while no significant correlation was found between HBV infection and previous history of blood transfusion, unprotected sexual behavior, and thalassemia.

**Conclusion:**

Based on the findings of this study, incarceration and drug abuse are the most important risk factors for acquiring HBV infection in this region. Modifying behavior, improving the individual education and expanding the HBV vaccination coverage may reduce the rate of infection in the region.

## Background

Hepatitis B virus (HBV) a member of hepadenaviridae family is the causative agent of hepatitis B infection. This infection remains a major global public health problem. In spite of the availability of a highly effective vaccine against hepatitis B infection, the overall burden of the disease remains enormous with over two billion people infected worldwide and about one million deaths annually [1,2].

It has been estimated that at the most, 33% of the infected people have evidence of clinical hepatitis, and depending on the age of infection, up to one third of infected patients become chronic carriers of hepatitis B surface antigen (HBsAg) [3].

Transmissions may occur through sexual intercourse, parenteral contact or from an infected mother to the baby at birth (4). HBV also shares similar characteristics to the HIV transmission, the virus has been detected in peripheral mononuclear cells, tissue of pancreas, kidney, skin, and spleen and of fluids like semen, breast milk, sweat, tears, urine and vaginal secretion [4,5].

The prevalence of hepatitis B infection varies in different parts of the world, ranging from less than 1% to 15%. In the Middle East, the endemicity is intermittent, with a carrier rate of 2% to 8% [6-9].

A systematic review on HBV infection in Iran revealed that prevalence of this infection is approximately 2.14% (range, 1.3% to 6.3%) across provinces [10]. The prevalence of HBV infection is higher among males than females in Iran [10]. Knowledge about the rate of hepatitis B infection and its related risk factors are necessary for implementating any preventive program. The present study was conducted to assess the prevalence and risk behaviors associated with HBV infection among high risk groups in Kohgiloyeh and Boyerahmad province, in Southwest of Iran. These high risk groups are the most vulnerable groups for acquiring HBV infection and have not been looked at in prior prevalence studies in the region.

## Methods

This descriptive cross-sectional study was carried out in Kohgiloyeh and Boyerahmad province, in southwest of Iran. A structured questionnaire containing demographics data such as age, gender, education level was used to collect the data. High risk populations for HBV infection including those who had a history of blood transfusion, history of intravenous drug use, history of incarceration, those with unsafe sexual activities, health care workers, patients on maintenance haemodialysis, hemophilic patients from main three townships of the province (Yasouj, Gachsaran, and Dehdasht) were the subjects of this study.

After getting approval from the ethics committee of Yasouj University of Medical Sciences, 5 ml of blood samples were obtained from each of 2009 participants including, 616 inmates (total inmates in three main prisons in the province), 158 injection drug users (those can be reached) 222 health care workers (based on the population of health care workers in the province), 49 thalassemic patients and from other high risk groups as presented in Table 1. Written informed consent for participation in the study was obtained from participants, or their parent or guardian, where participants were children. All sera were tested for antibodies to hepatitis B core antigen (HBc IgM, IgG) by enzyme-linked immunosorbent assay (ELISA, Dialab, Austria). Seropositive specimens were tested for HBsAg. HBsAg was determined using the Dialab-HBsAg ELISA Kit (Austria), which utilizes a sandwich immunoassay to detect HBsAg. All positive samples were tested twice for the confirmation of the results. Positive antibodies to hepatitis B core antigen were considered to define the presence of HBV infection and chronic infection was defined when both HBsAg and HBcAb (IgG) were present.

**Table 1:** Risk factors associated with HBV infection in high risk groups

**Risk factor**	**HBV positive**	**HBV negative**	**Total**	**P value**
History of imprisonment	13 (2.1%)	603	616	<0.05
History of using injecting drug	5 (3.16%)	153	158	<0.05
Transfusion	0 (0%)	22	22	>0.05
Needle stick	0 (0%)	222	222	>0.05
Thalassemia	0 (0%)	49	49	>0.05
Hemophilia	0 (0%)	3	3	>0.05
Unprotected sex activities	0 (0%)	16	16	>0.05
Others†	11 (1.19%)	912	923	>0.05

† Tattooing, history of surgery, dental practice, having HBV positive patients in the family.

Collected data were analyzed by SPSS version 17 software. The standard chi square test was used to assess the correlation of demographic and behavioral variables and HBV infection.

## Results

Two thousand and nine high risk people for HBV from three main townships of Kohgiloyeh & Boyerahmad providence were included in this study. Among them 802 (39.9%) were from Yasouj, 803 (40%) from Gachsaran and 404 (20.1%) were from Dehdasht. There were 1240 males (69.1%) and 555 females (30.9%). The mean age of the participants was 34.9 years (range, 2 to 82 years). The majority (39.6%) were in the 21–30 year age group.

The overall prevalence of HBV infection in this population was 1.2%. Considering the sex of participants, males were more susceptible to HBV infection. HBV infection was found to be more prevalent among those who were working in private sectors (3.8%) in comparison with state or health care workers. The prevalence of HBV infection was 3.2% among injection drug users. The highest rate of infection (4.2%) was seen among immigrant people while the highest rate of infection among native people was 1.2%. Regarding the place of residence, Yasouj (1.4%) had the highest rate of infection followed by Gachsaran (1.2%) and Dehdasht (0.65%). Rate of infection in inmates was 1.2% while none of hemophilia and thalassemia patients were found to be positive.

Significant associations were identified between HBV infection and sex, history of imprisonment, drug abuse, and place of residence (p<0.05). No statistically significant association was observed between HBV infection and history of needle stick (in health care workers) (p>0.05). Regarding the age of participants, 9.5% of subjects were between 1 to 20 years old, 39.6% between 21 to 30 years, 21.7% between 31 to 40 and the rest were above 40 years old. Although the highest rate of HBV infection (2.4%) was found in participants with less than 20 years old, but the differences between age and HBV infection was not significant (p>0.05). The risk factors associated with HBV infection in high risk groups are presented in Table 1. Multivariable analysis using logistic regression showed that history of imprisonment and drug abuse were independently associated with HBV infection. Table 2 shows the details of this correlation.

**Table 2:** Association between HBs antigen positivity and high risk behavior in high risk groups in Kohgiloyeh and Boyerahmad province, southwest of Iran

**High risk behavior**	**df**	**Sig.**	**Odd ratio**	**95.0% C.I. for EXP(B)**	
				**Lower**	**Upper**
History of drug use	1	.014	4.263	1.273	16.476
History of imprisonment	1	.043	2.170	1.364	8.814

## Discussion

Hepatitis B virus infection is a public health problem and a major cause of morbidity and mortality particularly in developing countries [8]. The world can be divided into three areas where the prevalence of chronic HBV infection is: high (>8%), intermediate (2-8%), and low (<2%) [8,9]. Most countries in the world are still considered intermediate to high endemicity for HBV infection [8,9]. In Iran the prevalence of hepatitis B varies between 1.3 to 6.3% in different areas of the country [10]. Because of lack of information about HBV infection in southwest of Iran the present study was conducted to look at the seroprevalence of HBV in high risk groups in this area.

A prevalence of 1.2% was found for HBV infection among the participant in this study. Comparing the findings of this study with other reports from the country, lower prevalence of HBV can be seen in this area [10,11]. Findings of this study demonstrated a relatively low prevalence of HBV in the region. Since the recruited subjects of this study are the selected high risk groups, therefore the rate of HBV in whole population of the district might be different. This low rate of HBV infection in the province might be connected to HBV vaccination coverage in this area. National levels of HBV1, HBV2 and HBV3 vaccination coverage in Iran have been reported to be 98.9%, 98.8% and 98.4% respectively. Vaccination coverage of HBV in Kohgiloyeh and Boyerahmad province is up to 99.5% [12].

In a recent study the overall prevalence of HBsAg positivity among general population in northeast of Iran was found to be 1.39% which is higher than the rate of infection in our study [13].

An unexpected observation in this study was that all positive HBV infections were males. There was no obvious explanation for the difference in gender, as a risk factor, for these viral infections. Having no infection in females might be due to the high risk groups which were evaluated in this study. Positive cases are all among those with a history of imprisonment or history of using injection drugs. More than 95% of these two groups were males. Furthermore, most of females participants in this study were either health care workers (with a well coverage of HBV vaccination), or those with history of tattooing, history of surgery, etc. and these factors are not as important as history of imprisonment or history of using injecting drug. In contrary to our findings, some studies demonstrated a lower prevalence of HBV infection in males than females [14].

Intravenous drug users are at higher risk for HBV infection, primarily through high risk sexual activities and sharing unsterile needles. The prevalence of HBsAg among male injecting drug users (IDUs) in Tehran, Iran, was reported to be 5.8% while in Zahedan, Southeast of Iran, rate of HBV in hospitalized injection drug users was found to be 19.3% [14]. In another study, the prevalence of HBV infection among hospitalized intravenous drug users in Ahvaz, southwest Iran, was 3.6% [15].

In our study the prevalence of HBV infection among injection drug users was 3.16% which is slightly lower than those reported from Tehran or other parts of the country [14].

Rate of HBV infection in prisoners in our study was 2.1% and a positive correlation was found between being in prison and seropositivity to HBV. This is again much lower than the rate of infection in incarcerations in other parts of the country. A study in south of Iran, in Bushehr province, reported a rate of 16.7% of HBV infection in prisoners [16].

In our study none of the hemophilic or thalassemic patients were positive for HBV infection. Repeated blood transfusions, as in thalessemia cases, are among the highest risk factors for HBV and HCV infection. In an interesting study in Bangladesh, the prevalence of transfusion-mediated viral infections in multi-transfused thalassemics individuals has been evaluated prior to initiate blood transfusion and after the subjects have received an average of 17 blood transfusions over a ten-month study period. The HBV and HCV markers have been significantly higher in post-transfused subjects as compared to pre-transfusion levels (for HBsAg; 19.0 vs. 7.1%) [17]. Correlation between thalessemia and hemophilia and HBV infection were insignificant in our study and this might be due to the limited number of cases in our study.

The proper screening of donated blood in blood banks, regarding HBV infection, and high coverage of vaccination of people who are afflicted by hemophilia, thalassemia might be contributed to the low rate of infection in these high risk groups.

Iranian blood transfusion organization (IBTO) is the only nationally qualified organization in Iran that performs blood transfusion procedures. This government-based organization provides its services free of charge. According to the most recent estimations, there are 23 blood donors per 1000 population. Only in 2005, the IBTO produced 1,600,000 units of blood for transfusion purposes. Approximately 96% of blood donations in Iran are collected from voluntary non-remunerated blood donors and the rest (4%) is donated as family replacement donation [18]. Khedmat et al., (2007) reported a rate of 0.487% for HBs antigen in Iranian blood donors [19].

The finding of this study showed that the people with a history of vaccination were infected by hepatitis B with a rate of 0.6% and non-vaccinated people with a rate of 2.3%. In current study, from all the subjects, 53% had a history of at least one dose of HBV vaccination. HBV infection was less prevalent (0.6%) in vaccinated individuals and there was a significant correlation between increasing number of vaccine doses and decreasing the rate of HBV infection.

## Conclusions

In conclusion, it is essential to assess the magnitude of the problem of HBV infection in each region of the country. Findings of the present study provided epidemiological features of hepatitis B and its risk factors in Kohgiloyeh and Boyerahmad province in Southwest of Iran. This information may help to restrain the spread of HBV infection in this and other similar settings in the country. The findings may also help the health authorities for better implementation of any prevention and control program.

## Competing interests

There are no financial or non-financial competing interests related to this manuscript to declare.

## Authors’ contributions

BS participated in study design, conducted the experiments and drafted the manuscript. AK collected the samples and conducted the experiments. OE and HN participated in sample collecting and conducting the experiments. MAT contributed in statistical analysis of the data. All authors read and approved the final manuscript.

## Pre-publication history

The pre-publication history for this paper can be accessed here:

http://www.biomedcentral.com/1471-2334/12/378/prepub
